# Identifying Mild Behavioral and Neurocognitive Impairment in Amyotrophic Lateral Sclerosis (MBNI‐ALS) Provides Key Prognostic Insights

**DOI:** 10.1111/ene.70171

**Published:** 2025-05-01

**Authors:** Myriam Spisto, Pasquale Moretta, Gianmaria Senerchia, Valentina Virginia Iuzzolino, Lucia Aruta, Elena Salvatore, Gabriella Santangelo, Luigi Trojano, Raffaele Dubbioso

**Affiliations:** ^1^ Department of Neurosciences, Reproductive Sciences and Odontostomatology University Federico II of Naples Naples Italy; ^2^ Department of Psychology University of Campania Luigi Vanvitelli Naples Italy; ^3^ Istituti Clinici Scientifici Maugeri IRCCS Neurological Rehabilitation Unit of Telese Terme Institute Benevento Italy; ^4^ Department of Advanced Biomedical Sciences Federico II University Naples Italy

**Keywords:** behavioral impairment, cognitive impairment, dementia, MCI, survival

## Abstract

**Background:**

Amyotrophic lateral sclerosis (ALS) is a multisystem neurodegenerative disease encompassing cognitive and behavioral impairments. The Revised Diagnostic Criteria for ALS‐frontotemporal spectrum disorder (ALS‐FTDS), while widely adopted, may overlook subtle impairments such as memory and visuospatial deficits, limiting their prognostic value.

**Objectives:**

This study aimed to apply the Mild Behavioral and Neurocognitive Impairment (MBNI) approach, adapted from other neurodegenerative diseases, to ALS patients and assess its prognostic utility for survival and disease progression.

**Methods:**

A prospective cohort of 201 ALS patients was evaluated between January 2018 and July 2024. Participants underwent comprehensive cognitive and behavioral assessments. The MBNI approach identified patients with mild cognitive impairment (MCI), mild behavioral impairment (MBI), or combined cognitive‐behavioral impairment (MCBI). Prognostic value was analyzed using Kaplan–Meier survival curves, Cox proportional hazards models, and logistic regression for disease progression.

**Results:**

Mild cognitive and/or behavioral impairments were detected in 67% of patients classified as cognitively normal by ALS‐FTDS criteria. At a median follow‐up of 15 months, these patients showed shorter tracheostomy‐free survival (all *p* < 0.005). MCI (HR5.3; CI 1.10–25.41; *p* = 0.038) and frontotemporal dementia (HR6.2; Confidence Interval: 1.34–28.40; *p* = 0.019) independently predicted poor outcomes. Logistic regression confirmed that MCBI and frontotemporal dementia were associated with rapid progression (both *p* < 0.019).

**Conclusion:**

The MBNI approach enhances the detection of mild cognitive and behavioral impairments in ALS, providing prognostic insights and improving stratification over the Revised Diagnostic Criteria for ALS‐FTDS. This framework supports personalized care and the design of clinical trials targeting early disease stages.

## 1 Introduction

The traditional view of Amyotrophic Lateral Sclerosis (ALS) has evolved from being a purely motor neurodegenerative disorder to a multisystem disease encompassing non‐motor symptoms. Cognitive and behavioral changes [1], autonomic dysfunction [2], sensory alterations [3], and sleep disturbances [4] are now recognized as integral to the disease.

Cognitive impairments, particularly executive dysfunctions, and behavioral disturbances may manifest early in ALS [5] and are linked to more rapid disease progression, reduced survival, and increased caregiver burden [6, 7].

Several studies proposed that motor and cognitive manifestations of ALS decline in parallel, with cognitive impairment correlating with revised ALS Functional Rating Scale (ALSFRS‐R) scores [8, 9]. Cross‐sectional observations further support this association, suggesting that cognitive dysfunction may serve as a marker of disease severity [10, 11]. Additionally, cognitive and behavioral impairments have been linked to more rapid disease progression and shorter survival [8, 12, 13].

The Edinburgh Cognitive and Behavioral ALS Screen (ECAS) [14] remains a valuable tool for detecting cognitive dysfunction in ALS. However, it may not fully capture subtle impairments of memory and visuospatial functions, which hold potential prognostic value [15].

The widely adopted Revised Diagnostic Criteria for ALS‐frontotemporal spectrum disorder (ALS‐FTDS) criteria [16], introduced by Strong and colleagues [16], provide a structured framework for classifying cognitive and behavioral symptoms in ALS, including cognitively normal (CN), Cognitive Impairment (CI), Behavioral Impairment (BI), Combined Cognitive‐Behavioral Impairment (CBI), and Frontotemporal Dementia (FTD). Nevertheless, these criteria [16] may overlook early cognitive changes that could be detected by more sensitive tools and could serve as markers of disease progression. Indeed, the concept of cognitive impairment introduced by Strong et al. [16] captures intermediate cognitive dysfunction focused on executive and language deficits but does not fully encompass other milder impairments.

Frameworks like mild cognitive impairment (MCI) [17, 18] and mild behavioral impairment (MBI) [19, 20, 21] in other neurodegenerative diseases aim to identify early and mild deficits before dementia onset. Applying MCI/MBI‐like criteria for ALS could capture these subtler early changes, rather than redefining the Strong cognitive classification itself. This idea would be in line with the recent introduction of Mild Motor Impairment as a prodromal state in ALS [22], which emphasizes the need to extend such principles to the cognitive and behavioral domains.

Early recognition of mild cognitive and behavioral changes may enable the timely initiation of pharmacological treatments targeting non‐motor symptoms, thereby improving clinical management and enhancing patients' and caregivers' quality of life, particularly for genetic forms of ALS [23]. Detecting these early changes could also contribute to identifying significant predictors of disease progression useful to inform therapeutic decision‐making and the design of clinical trials.

We aimed to identify Mild Behavioral and Neurocognitive Impairments (MBNI) in ALS by applying criteria for MCI and MBI, and to evaluate the potential prognostic value of this approach. We hypothesize that recognizing early impairments will offer a more comprehensive assessment of cognitive health, enhance risk stratification, and improve understanding and clinical management of the disease's non‐motor spectrum.

## 2 Participants

Between January 2018 and July 2024, 201 patients with amyotrophic lateral sclerosis (ALS) were consecutively recruited at the ALS Clinic Centre Federico II University Hospital. Patients were followed until September 2024, through both onsite visits and remote telephone follow‐ups conducted every 3 months. All patients met the revised El Escorial diagnostic criteria of possible, probable, probable laboratory‐supported, or definite ALS [24]. At study entry, all patients underwent a comprehensive evaluation, including neurological history, neurophysiological assessment, and clinical, cognitive/behavioral, and mood examinations. ALS patients also underwent genetic screening for the four most common genes associated with ALS: *C9orf72*, *FUS*, *SOD1*, and *TARDBP*. All clinical examinations were performed by experienced neurologists (R.D., G.S. and V.V.I.), and the site of disease onset and disease duration were recorded. Disease severity was assessed using the ALSFRS‐R [25], while respiratory function was assessed through spirometry with the patient sitting upright [26]. We computed the ALSFRS‐R median monthly decline (∆ALSFRS‐R) using the following formula [27]: (48 − ALSFRS‐R score at baseline assessment)/(months from onset to baseline assessment). In addition, disease progression was classified according to King's staging system, which evaluates ALS progression across four stages based on the number of regions involved and the need for feeding or respiratory assistance [28].

The research was conducted in accordance with the Declaration of Helsinki, and all participants provided their written informed consent prior to inclusion in the study.

## 3 Cognitive and Behavioral Evaluation

Following the clinical assessment, all participants underwent a comprehensive neuropsychological battery to evaluate their cognitive and behavioral profiles. The cognitive battery, administered by a neuropsychologist with ALS expertise (M.S.), included standardized Italian tests assessing executive functions, language, visuospatial abilities, and memory, validated on a large Italian population. As part of this assessment, the ECAS [14], a widely used multi‐domain screening tool for ALS patients and translated into several languages, was administered. However, since ECAS is primarily a screening tool, a more extensive neuropsychological evaluation was performed to provide a detailed assessment of key cognitive domains (See Supporting Information). The neuropsychological battery was designed to comprehensively assess cognitive functions typically affected in ALS, with a particular focus on executive functions, language, and visuospatial abilities. Multiple tests were used for executive functions to capture distinct subdomains (e.g., cognitive flexibility, working memory, inhibition). To ensure robustness and avoid overestimation of impairments, a diagnosis of executive dysfunction required deficits in at least two independent tests within the same domain.

In addition, behavioral changes typically associated with ALS were evaluated through short semi‐structured [16] and structured [21, 29] interviews with caregivers. Furthermore, patients were assessed for their autonomy in basic daily living activities (ADL) and instrumental activities of daily living (IADL) [30].

These evaluations allowed classifying patients using an approach commonly applied in other neurodegenerative diseases [18, 19, 31]. This approach identifies patients with MCI as those with cognitive impairment without significant functional decline, defined by scores at least two standard deviations below the expected mean, adjusted for age and education. In line with the MCI diagnostic criteria [32], cognitive decline was identified through reports from the patient, their informant, or clinical observation. All MCI cases included in the study reflected a change in cognitive performance, ensuring consistency with the established criteria.

MCI was classified into two subtypes: Single‐Domain, involving deficits in two non‐overlapping tests within a single cognitive domain, and Multiple‐Domain, characterized by impairments in at least one test across two or more cognitive domains [32].

Additionally, mild MBI was identified in patients showing early behavioral changes using a structured questionnaire: the Mild Behavioral Impairment Checklist [21, 33].

Behavioral impairment was assessed according to ISTAART‐AA criteria for MBI [20, 21], excluding mood symptoms such as depression and anxiety, which were evaluated separately when possible. We prioritized core MBI domains (e.g., apathy, disinhibition), minimizing the risk of confounding emotional dysregulation with mood disturbances due to disease progression. Patients demonstrating concurrent early cognitive deficits and behavioral changes were classified as having mild cognitive and behavioral impairment (MCBI). Lastly, patients without any cognitive or behavioral impairment were classified as having normal cognition (NC).

We also evaluated the distribution of early cognitive and behavioral changes at baseline in reference to the Strong classification system [16], which categorizes patients as CN, CI, BI, or CBI.

Patients fulfilling diagnostic criteria for FTD in all its variants, as well as those presenting with dementia not typical of FTD, were included under the umbrella term FTD‐dem [16]. For further details on these dementia criteria and the cognitive evaluation, please refer to the Supporting Information and Table 1.

**TABLE 1:** Comparison between the STRONG classification and MBNI approach for cognitive and behavioral impairment in ALS.

Strong classification		MBNI approach	
Category	Description	Category	Description
ALS‐Cognitively Normal (CN)	No cognitive or behavioral impairments	ALS‐Normal Cognition (NC)	No mild cognitive or mild behavioral impairments
ALS‐Cognitive Impairment (CI)	Deficits in executive function (including social cognition) or language dysfunction, affecting two non‐overlapping tests, or a combination of both.	ALS‐Mild Cognitive Impairment (MCI)	Cognitive impairment without significant functional decline defined by scores at least two standard deviations below the expected mean, adjusted for age and education. Evaluated cognitive domains include executive functions, memory, visuospatial abilities, and language – *MCI Single‐Domain*: deficits in two non‐overlapping tests within a single cognitive domain – *MCI Multiple‐Domain*: deficits in at least one test across two or more cognitive domains
ALS‐Behavioral Impairment (BI)	The identification of apathy with or without other behavior change OR meeting at least two non‐overlapping supportive diagnostic features from the Rascovsky criteria [34]	ALS‐Mild Behavioral Impairment (MBI)	Changes in behavior such as apathy, affective/emotional dysregulation, impulse control, social inadequacy, and abnormal thoughts/perceptions using the Mild Behavioral Impairment Checklist (MBI‐C)
ALS‐Cognitive and Behavioral Impairment (CBI)	Patients who meet the criteria for both ALS‐CI and ALS‐BI	ALS‐ Mild Cognitive and Behavioral Impairment (MCBI)	Patients who meet the criteria for both ALS‐MCI and ALS‐MBI
ALS‐Frontotemporal Dementia (FTD)	Patients who meet the criteria for frontotemporal dementia	ALS‐Frontotemporal Dementia (FTD)	Patients who meet the criteria for frontotemporal dementia

Clinically relevant depression and anxiety symptoms were evaluated through the Hamilton Depression Scale (HDS) and Beck Anxiety Inventory (BAI) [35].

## 4 Statistical Analysis

All statistical analyses were performed using SPSS 29.0 (IBM Corp., Armonk, NY, USA), with a significance threshold set at *p* < 0.05. Descriptive statistics summarized demographic, clinical, and neuropsychological characteristics. Given the non‐parametric distribution of the data, as confirmed by the Shapiro–Wilk test, non‐parametric methods were employed. Group comparisons were conducted using Mann–Whitney *U*, Kruskal–Wallis, and Chi‐square tests, stratified by the Strong and MBNI classifications, with Bonferroni correction applied for multiple comparisons. Kaplan–Meier survival analysis assessed tracheostomy‐free survival, with differences evaluated via the log‐rank test. Cox proportional hazards models, adjusted for key clinical covariates, estimated hazard ratios. Additionally, binary logistic regression examined disease progression, incorporating cognitive classifications as independent variables.

## 5 Results

### 5.1 Clinical Characteristics as a Function of Cognitive and Behavioral Impairments

Characteristics of the sample, as well as their distribution based on the two cognitive classification systems, are detailed in Table 2.

**TABLE 2:** Clinical characteristics of patients according to the two cognitive classifications.

	Strong classification						MBNI approach						All population
	CN (92)	CI (36)	BI (21)	CBI (22)	FTD‐dem (30)	*p* value	NC (30)	MCI (31)	MBI (43)	MCBI (67)	FTD‐dem (30)	*p* value	*N* (201)
Age (years; median, IQR)	61.5 (52.75–71)	66 (60.75–72.25)	61 (54–67)	68.5 (62–72.5)	68 (65.25–74.75)	**0.003**	59 (50.25–68)	65.0 (59–72)	61 (53–69.5)	67 (61.5–73)	68 (65.25–74.75)	**< 0.001**	65 (58–72)
Education (years; median, IQR)	13.00 (8–16)	11 (5–13.25)	13.00 (8–17)	8.00 (5–11)	8.00 (5–12.75)	**0.001**	13 (11–17)	11 (8–13)	13 (8–16.5)	8 (5–13)	8 (5–12.75)	**< 0.001**	13.00 (8–25)
Tracheostomy free survival time (months; median, IQR)	15 (5–26.3)	10 (5.5–23)	15 (6–24)	15 (6.3–28)	9 (5–19)	0.549	15 (4.3–30.5)	11 (6.3–21.8)	16 (7.5–28)	13 (5–25)	9 (5–19)	0.385	13 (5–25)
Diagnostc delay (months; median, IQR)	9.00 (7–19)	11.50 (6–21.5)	14 (9–21)	12 (8.5–19)	10 (6–15.3)	0.644	9 (6.3–19.8)	9 (7.5–16)	11 (7–20)	12 (7–20.5)	10 (6–15.3)	0.875	11 (7–19)
FVC (%; median, IQR)	90.5 (77–101)	81.00 (61–92)	90.00 (83–103)	70.50 (54–91)	76.00 (45–96)	0.039	95 (83.5–104.5)	82 (73.8–96.3)	88.5 (80–101)	83 (59–96)	76 (44.8–96)	0.025	86.5 (67.8–99)
ALSFRS‐R (median, IQR)	40.0 (34.75–43.0)	38.5 (31.25–41.25)	34.0 (29–41)	33.5 (31–37)	30.5 (24.75–37.5)	**< 0.001**	41 (36–42.8)	40 (37–43)	40 (32–42)	35 (28–39.5)	30.5 (24.75–37.5)	**< 0.001**	37 (30.5–42)
Rate of progression (median, IQR)	0.51 (0.29–1.22)	0.81 (0.51–1.38)	0.61 (0.35–1.2)	0.80 (0.7–1.41)	1.18 (0.65–2)	0.02	0.50 (0.32–1.3)	0.73 (0.26–1.46)	0.51 (0.3–0.83)	0.79 (0.44–1.52)	1.18 (0.65–2)	0.009	0.72 (0.37–1.39)
Male (*n*, %)	53 (57.6%)	27 (75%)	15 (71.43%)	17 (77.27%)	16 (53.33%)	0.128	19 (63.33%)	19 (63.33%)	26 (60.47%)	48 (71.6%)	16 (53.3%)	0.479	128 (63.7%)
BULBAR onset (*n*, %)	21 (22.83%)	9 (25.00%)	3 (14.29%)	3 (13.64%)	8 (26.7%)	0.692	5 (16.67%)	13 (41.94%)	6 (13.95%)	12 (17.91%)	8 (26.67%)	0.04	44 (21.9%)
Genetic testing, positive/tested (*n*, %)	8/85 (9.4%)	4/34 (11.8%)	0/18 (0%)	0/20 (0%)	2/27 (7.4%)	0.360	3/30 (10%)	1/29 (3.4%)	2/36 (5.6%)	6/62 (9.7%)	2/27 (7.4%)	0.817	14/184 (7.6%)
C9orf72 positive (*n*, %)	3/85 (3.5%)	3/34 (8.8%)	0/18 (0%)	0/20 (0%)	2/27 (7.4%)	0.391	1/30 (3.3%)	1/29 (3.4%)	1/36 (2.8%)	3/62 (4.8%)	2/27 (7.4%)	0.912	8/184 (4.4%)

*Note:* Comparisons between the groups were performed using the non‐parametric Kruskal–Wallis test, while frequencies were compared using the chi‐squared test. Values in bold type indicate significant *p* values after Bonferroni correction (*p* < 0.0056). Genetic testing was performed for the four main ALS‐associated genes: C9orf72 (hexanucleotide repeat expansion), SOD1 (Superoxide Dismutase 1), TARDBP (TAR DNA‐binding protein), and FUS (Fused in Sarcoma). ‘Genetic Testing, Positive/Tested’ indicates the number and percentage of patients with a pathogenic variant in any of these genes, while ‘C9orf72 Positive’ refers specifically to patients carrying the C9orf72 repeat expansion. The tracheostomy‐free survival time is calculated as the duration from the time of testing to the occurrence of either invasive ventilation (tracheostomy) or death.

Abbreviations: BI = behavioral impairment, CBI = combined cognitive and behavioral impairment, CI = cognitive impairment, CN = cognitively normal, FTD‐dem = frontotemporal dementia syndrome and other dementias, MBI = mild behavioral impairment, MBNI = mild behavioral and neurocognitive impairment, MCBI = mild combined cognitive and behavioral impairment, MCI = mild cognitive impairment, NC = normal cognition.

**FIGURE 1:**
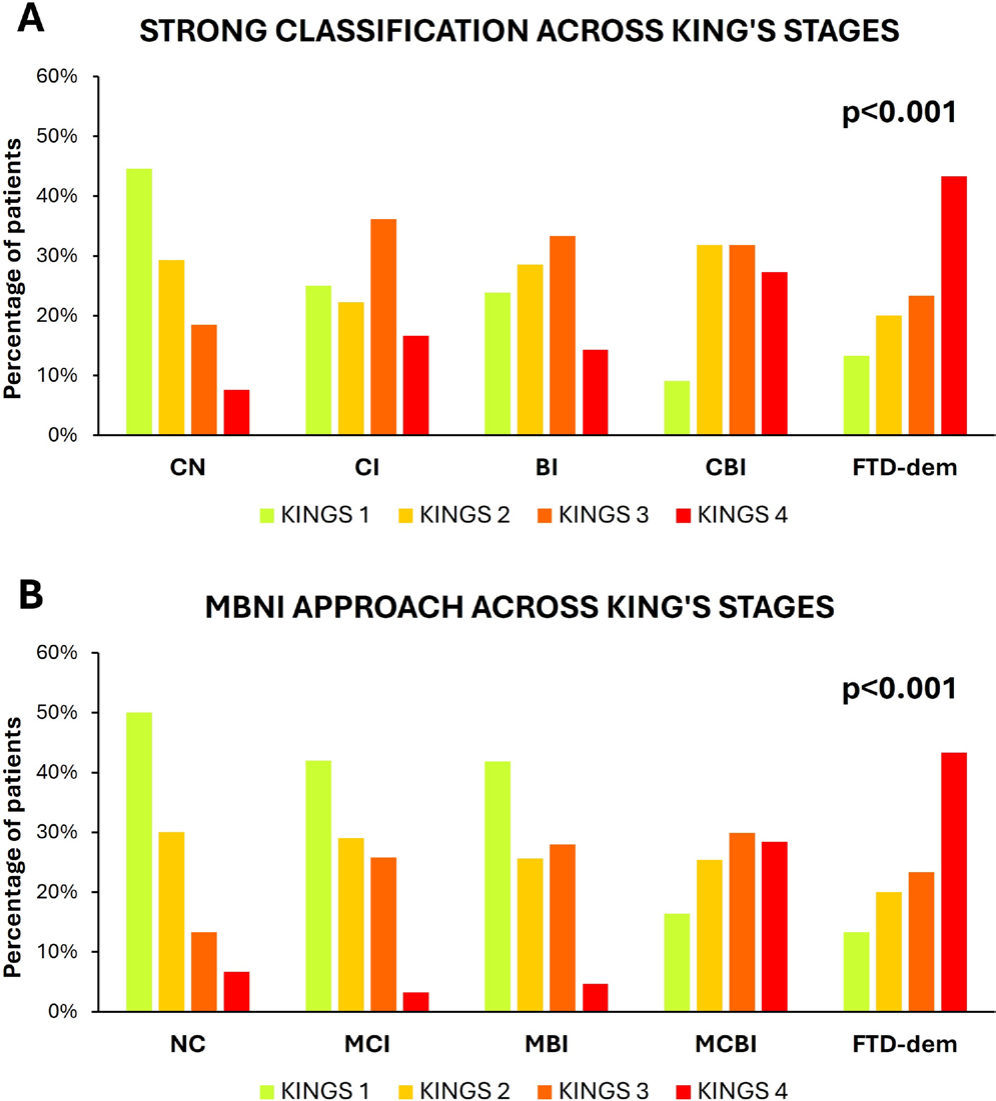
Cognitive and behavioral impairments across king's clinical stages using strong classification and MBNI approach. Frequency of cognitive and behavioral impairments across King's clinical disease stages, comparing Strong classification (A) and the MBNI approach (B). Chi‐squared tests were used to determine significance (*p* < 0.05). BI = behavioral impairment, CBI = combined cognitive and behavioral impairment, CI = cognitive impairment, CN = cognitively normal, FTD‐dem = frontotemporal dementia syndrome and other dementias, MCBI = mild combined cognitive and behavioral impairment, MCI = mild cognitive impairment, MBI = mild behavioral impairment; NC = normal cognition.

In more severe King's clinical stages, both the Strong classification and our approach showed a lower prevalence of CN and NC patients (Strong: *χ* ^2^ = 23.22, *p* < 0.001; MBNI: *χ* ^2^ = 8.65, *p* = 0.034), and a higher prevalence of FTD (Strong and MBNI: *χ* ^2^ = 17.51, *p* < 0.001). For intermediate categories, according to the Strong classification, CI, BI, and CBI were more frequently observed in patients with advanced disease stages (Figure 1A), whereas according to the MBNI approach, the proportion of MCI and MBI was higher in earlier stages, and MCBI was more prevalent in later stages (Figure 1B, *p* = 0.012).

According to both cognitive classifications, age significantly increased from cognitively normal to FTD‐dem (*p* = 0.003 in Strong, *p* < 0.001 in MBNI), and educational attainment decreased across cognitive stages, with FTD‐dem patients having the lowest median levels. FTD‐dem patients consistently showed lower FVC and ALSFRS‐R values compared to CN patients in both classifications. See Table 2 for further details.

### 5.2 Distribution of MBNI With Reference to Strong Classification

In the Strong classification, 92 patients (46%) were classified as CN, 36 patients (18%) as CI, 21 patients (10%) as BI, and 22 patients (11%) as showing CBI.

The present approach identified a relevant number of patients with early cognitive and behavioral impairments. In our cohort, 30 patients (15%) were classified as having NC, 31 patients (15%) as MCI, 43 patients (21%), and 67 patients (33%) as having MCBI. For both classifications, the FTD‐dem group included 30 patients (15%).

**FIGURE 2:**
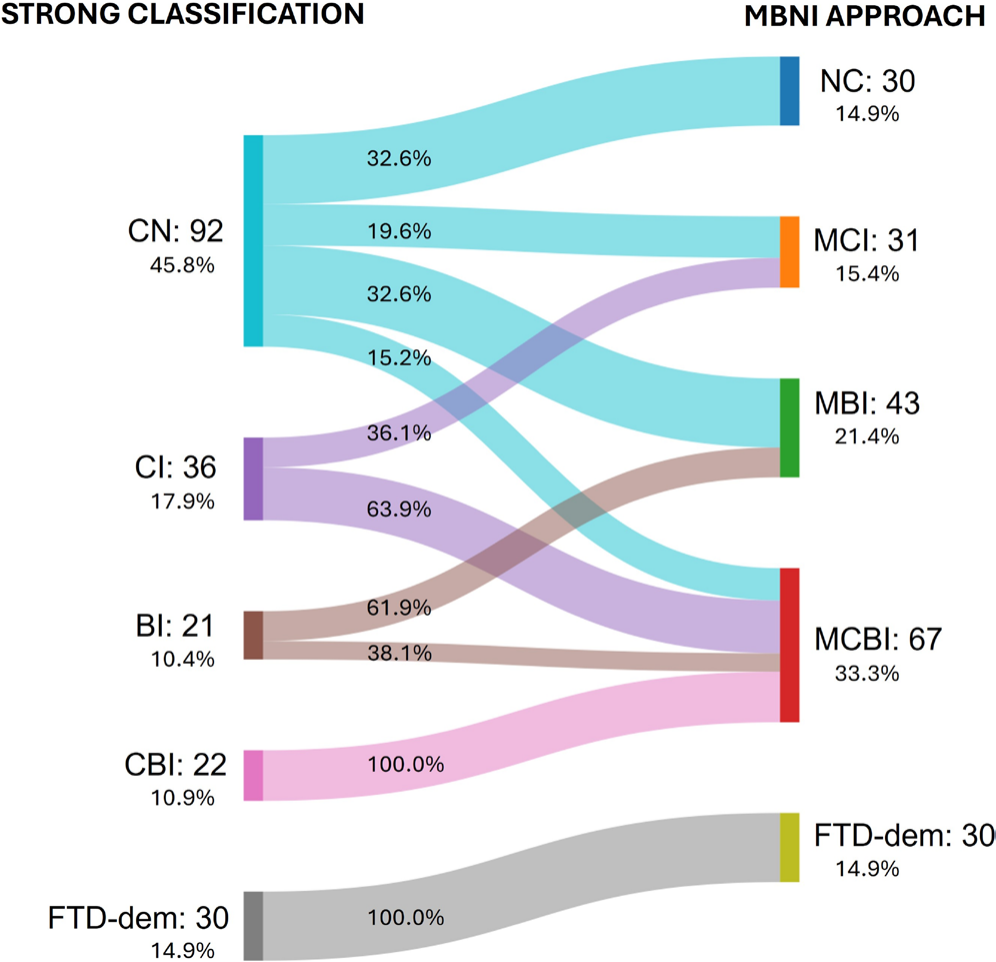
Sankey Diagram Comparing Patient Transitions Between Strong classification and MBNI approach for Cognitive and Behavioral Impairments in ALS. Sankey diagram illustrates the distribution of ALS patients across cognitive and behavioral impairment categories, comparing the Strong classification on the left with the MBNI approach on the right. Patients classified as cognitively normal (CN) in the Strong system are distributed across normal cognition (NC), mild cognitive impairment (MCI), mild behavioral impairment (MBI), and mild combined cognitive and behavioral impairment (MCBI) in the MBNI approach. Similarly, cognitive impairment (CI), behavioral impairment (BI), and combined cognitive and behavioral impairment (CBI) in the Strong classification transition primarily to MCI, MBI, and MCBI in the MBNI approach. Patients with frontotemporal dementia and other dementias (FTD‐dem) remain in the same category across both classifications. Thicker flows indicate a larger number of patients transitioning between categories.

The Sankey diagram shows how most patients classified as CN under the Strong criteria showed indeed mild cognitive and behavioral impairments according to the proposed framework (Figure 2).

Among the 62 out of 92 patients (67%) classified as CN according to the Strong criteria, but with mild cognitive and/or behavioral alterations, specific cognitive domains were affected as follows: one patient exhibited impairment in a single memory domain, two patients showed deficits in a single visuospatial domain, and notably, 29 patients demonstrated impairments across multiple cognitive domains (Figure S1A). This underscores the high prevalence of multiple cognitive deficits within this subgroup.

Most patients classified with multidomain MCI (*n* = 29) had impairments in the most commonly affected domains in ALS: 12 patients (41.4%) in executive and visuospatial domains, 7 patients (24.1%) in executive and memory domains, whereas 1 patient (3.4%) showed defects in executive, memory, and visuospatial domains. Less common combinations were: executive, language, and memory deficits (2 patients, 6.9%); executive, language, and visuospatial deficits (2 patients, 6.9%); executive, memory, language, and visuospatial deficits (2 patients, 6.9%). Further combinations of deficits, not affecting the executive domain, were memory and visuospatial deficits (1 patient, 3.4%); language and visuospatial deficits (1 patient, 3.4%); language, memory, and visuospatial deficits (1 patient, 3.4%).

Several patients classified as CN according to the Strong criteria (Figure S1B) showed affective or emotional dysregulation, impulse control problems, and apathy. Additionally, social inadequacy and abnormal thoughts or perceptions were observed in a minority of patients. A detailed characterization of cognitive and behavioral impairments in the CN group is provided in Table S2.

In a subgroup of 75 patients assessed at the time of cognitive testing, none had severe depressive symptomatology, 3 patients (4.0%) had moderate depression symptoms, 23 patients (30.7%) had mild symptoms, and 49 patients (65.3%) showed no signs of depression. Regarding anxiety, 3 patients (4.0%) experienced severe symptomatology, 12 (16.0%) had moderate symptoms, 16 (21.3%) had mild symptoms, and 44 (58.7%) reported minimal or no anxiety. No correlation was found between depression or anxiety symptomatology and either the cognitive classifications adopted or the disease clinical staging.

### 5.3 Survival Outcomes in ALS Patients Based on Cognitive and Behavioral Profiles

**FIGURE 3:**
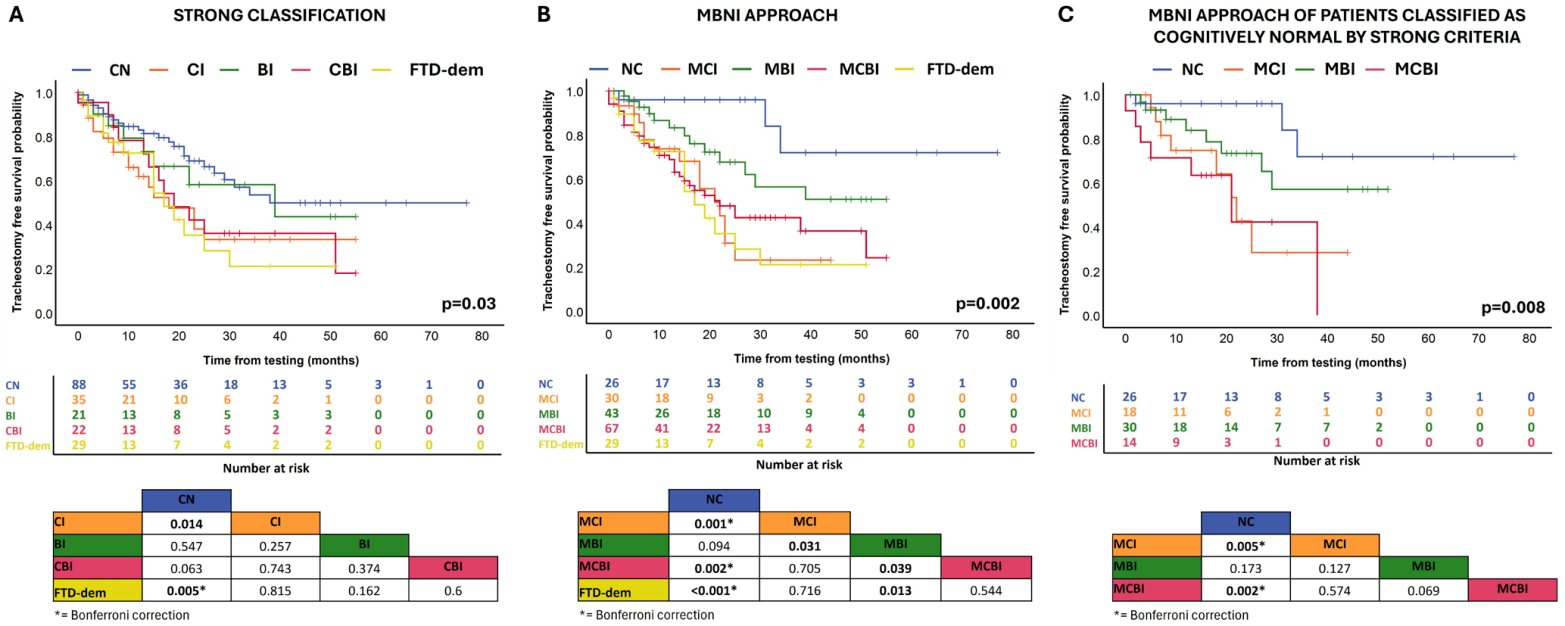
Survival analysis according to MBNI approach, Strong criteria, and in the subgroup of patients classified as cognitively normal according to Strong criteria. In Panel A, the Kaplan–Meier analysis reveals that, according to the Strong classification, only patients with FTD‐dem (frontotemporal dementia and other dementias) demonstrate a significantly poorer prognosis compared to cognitively normal (CN) individuals. In Panel B, the Kaplan–Meier curve based on the MBNI approach shows a distinct separation of the MCI (mild cognitive impairment), MCBI (mild combined cognitive and behavioral impairment), and FTD‐dem groups from those with normal cognition (NC), indicating a worse prognosis for these classes. In Panel C, a subgroup analysis of patients classified as CN under the Strong criteria indicates that those with MCI and MCBI exhibit a poorer prognosis compared to NC individuals. Beneath each survival curve, the number of patients at risk for each subgroup is displayed, accompanied by a table summarizing the results of multiple comparisons. Significant *p* values are highlighted in bold, and asterisks mark those that remain significant after Bonferroni correction.

Kaplan–Meier survival curves indicated differences in survival outcomes based on cognitive and behavioral classifications defined by Strong criteria and the present approach (Figure 3). For the Strong classification, there was a significant overall effect (*p* = 0.03). Pairwise analyses showed reduced survival in patients with CI compared to those without (CN) and indicated a trend toward poorer outcomes for the CBI group in comparison to CN. Additionally, the frontotemporal dementia group (FTD‐dem) demonstrated significantly worse survival outcomes compared to CN (*p* = 0.005).

According to our approach, the global comparison indicated a significant overall effect too (*p* = 0.002). Pairwise analyses demonstrated that MCI, MCBI, and FTD‐dem consistently showed worse survival compared to NC. Notably, MBI patients had worse survival than NC but fared better than those with MCI, MCBI, and FTD‐dem.

A further survival analysis focused on patients classified as CN according to the Strong criteria and as NC, MCI, or MBI/MCBI under the present classification (Figure 3C). This analysis yielded a significant global effect (*p* = 0.008), highlighting significant differences in survival outcomes. Specifically, MCI and MCBI groups displayed worse prognoses compared to the NC classification (all *p* < 0.005).

The univariate Cox analysis highlighted several factors significantly associated with an increased mortality risk in ALS patients (Supplementary Table 3), including older age (*p* < 0.001), bulbar onset (*p* < 0.001), fewer years of education (*p* = 0.001), more rapid functional decline (*p* < 0.001), shorter disease duration (*p* < 0.001) and advanced disease stage as reflected by King's stage (*p* < 0.001).

In terms of cognitive and behavioral impairments, the present approach revealed that patients with MCI had a significantly increased risk of death (*p* = 0.003), as did those with MCBI (*p* = 0.006), while FTD‐dem was strongly associated with poorer survival (*p* = 0.002). Similarly, the Strong classification system demonstrated that CI (*p* = 0.017) and FTD‐dem (*p* = 0.009) were linked to higher mortality, with CBI showing a borderline trend toward a worse prognosis (*p* = 0.069); see Table S3.

After adjusting for age, education level, diseases onset, progression's rate, disease duration, and King's stage, the multivariate survival analysis indicated that patients classified under the present approach with MCI had a HR of 5.3 (*p* = 0.038), suggesting a significantly increased risk of mortality compared to CN patients. Furthermore, individuals classified as FTD‐dem also exhibited a notable risk of increased mortality, with an HR of 6.2 (*p* = 0.019). Conversely, patients categorized under the Strong classification did not demonstrate statistically significant hazard ratios for mortality risk (Table 3).

**TABLE 3:** Multivariate Cox Regression analysis of clinical and cognitive variables predicting survival in ALS patients.

Variable	Multivariate		
	Hazard ratio	95% confidence interval	*p* value
MBNI approach			
Age (years)	1.01	0.99–1.04	0.296
Onset (reference: Bulbar)	0.78	0.44–1.38	0.392
Education (years)	0.94	0.89–1.00	**0.034**
Disease duration (months)	0.92	0.89–0.94	**< 0.001**
Rate of progression	0.91	0.74–1.14	0.422
King's stages	1.67	1.26–2.21	**< 0.001**
NC	Reference		
MCI	5.28	1.10–25.41	**0.038**
MBI	4.01	0.82–19.68	0.088
MCBI	4.52	1.00–20.41	0.050
FTD‐dem	6.17	1.34–28.40	**0.019**
Strong classification			
Age (years)	1.02	1.00–1.04	0.089
Onset (reference: Bulbar)	0.73	0.41–1.30	0.279
Education (years)	0.94	0.89–1.00	**0.037**
Disease Duration (months)	0.92	0.89–0.94	**< 0.001**
Rate of Progression	0.94	0.75–1.17	0.575
King's Stage	1.60	1.21–2.11	**0.001**
CN	Reference		
CI	1.63	0.85–3.12	0.139
BI	1.66	0.72–3.82	0.236
CBI	1.10	0.51–2.38	0.800
FTD‐dem	1.06	0.51–2.18	0.885

*Note:* In bold significant *p* < 0.05.

Abbreviations: ALSFRS‐R: Amyotrophic Lateral Sclerosis Functional Rating Scale—Revised; BI: behavioral impairment; CBI: cognitive and behavioral Impairment; CI: cognitive impairment; CN: cognitively normal; FTD‐dem: frontotemporal dementia and other dementias; MBI: mild behavioral impairment; MCBI: mild cognitive and behavioral impairment; MCI: mild cognitive Impairment; NC: normal cognition.

### 5.4 Impact of Mild Cognitive and Behavioral Impairments on Disease Progression

**FIGURE 4:**
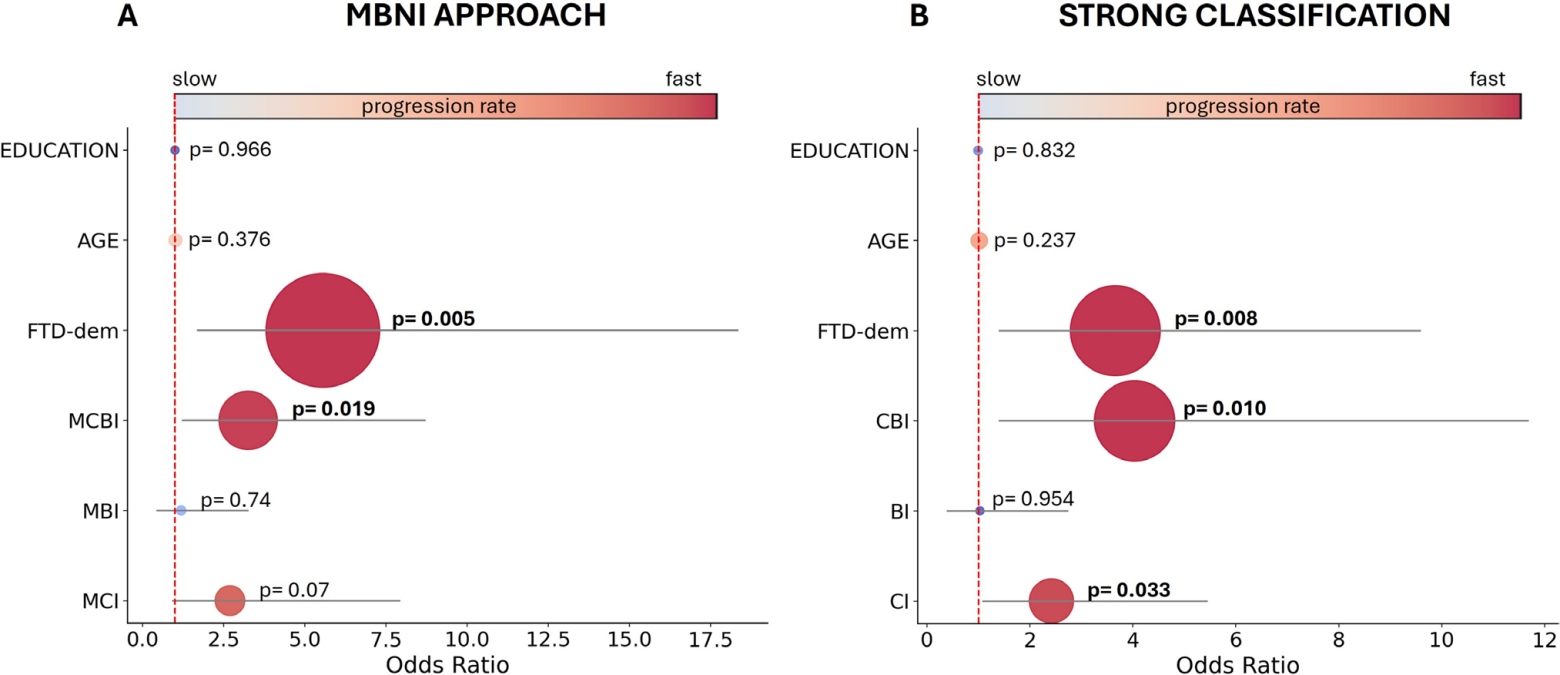
Binary logistic regression for disease progression rate. The plots display the odds ratio (OR) for each variable on the horizontal axis, based on cognitive classification: MBNI on the left (A) and Strong on the right (B). Values greater than 1 indicate a higher likelihood of disease progression, while values less than 1 suggest a lower likelihood. Each bubble represents a variable, with the size of the bubble reflecting its statistical significance (larger bubbles indicate smaller *p* values). The color of the bubble corresponds to the rate of progression: Red shades indicate fast progression, while blue shades represent slow progression. The horizontal bars extending from each bubble show the 95% confidence intervals. The exact *p* value is displayed next to each bubble, with *p* values below 0.05 (in bold) indicating significant associations.

Binary logistic regression analysis revealed that cognitive and behavioral classification systems are significant predictors of disease progression in ALS patients, even after adjusting for age and education level in both classification frameworks (Figure 4).

Within the MBNI classification system, patients identified with MCBI exhibited a significantly higher risk of disease progression compared to patients with NC (*p* = 0.019). Furthermore, those diagnosed with FTD‐dem faced an even greater risk of rapid disease progression (*p* = 0.005), Figure 4A.

Similarly, under the Strong classification system, patients classified as CI demonstrated a significantly increased likelihood of disease progression compared to CN patients (*p* = 0.033). Additionally, individuals with CBI and those with FTD‐dem showed markedly elevated risks of disease progression, with odds ratios of 4.04 (*p* = 0.010) and 3.68 (*p* = 0.008), respectively (Figure 4B).

## 6 Discussion

Our study demonstrates that the application of a specific diagnostic approach highlighted mild cognitive and behavioral disorders in patients with ALS that are often overlooked by traditional criteria. Notably, the MBNI approach significantly reduced the proportion of patients classified as cognitively normal—from 46% under the Strong criteria to 15% underscoring the importance of detecting subtle yet clinically significant changes. These changes carry prognostic weight, as patients classified as normal by Strong criteria but showing symptoms under MBNI had worse outcomes, highlighting crucial clinical implications. Milder forms of cognitive and behavioral impairment, such as MCI and MBI, were more prevalent during early ALS stages, while severe forms, including MCBI and FTD‐dem, dominated advanced stages. These findings highlight that MBNI can be present since early stages of ALS, whereas according to Strong classification, CI, BI, and CBI emerge in later stages. These findings suggest a gradual progression of cognitive and behavioral changes throughout ALS, with MBNI capturing patients across both early and late stages.

The mean delay between symptom onset and diagnosis was consistent across clinical stages, suggesting a link between cognitive/behavioral changes and motor decline. Cognitive alterations may begin early, coinciding with motor cortex involvement, and could reflect early pathology in cognitive regions such as the prefrontal and temporal cortices. Predictable demographic differences across King's stages, with older patients at advanced stages, align with the epidemiology of FTD, which increases with age [36]. Patients with advanced impairments had fewer years of education, reinforcing the role of cognitive reserve in modulating the impact of brain damage, as observed in Alzheimer's disease [37], FTD [38], and ALS [34].

Interestingly, evidence suggests that cognitive impairments, particularly in executive and visuospatial domains, may influence motor function, including gait dynamics and fall risk [39]. While our study did not directly assess these aspects, previous research [40] has indicated that MCI in ALS can be associated with increased gait variability and a higher risk of falls.

Another interesting aspect of the MBNI approach is its potential for assessing cognitive changes over time. To date we have no long‐term cognitive longitudinal data, but current studies report heterogeneous findings on longitudinal cognitive changes in ALS. Some indicate that cognitive and behavioral impairments can evolve over time [9] even in early disease stages [5], with a subgroup showing decline across all domains, especially memory and visuospatial functions [15]. Conversely, other studies suggest that cognitive functions remain stable throughout the disease course [12]. This variability underscores the need for a more comprehensive classification system, like the one proposed here, which can prove very useful in longitudinal studies.

Beyond understanding impairments, MBNI offers substantial implications for clinical trials. Identifying patients in earlier stages of decline facilitates better risk stratification and improved targeting of non‐motor symptoms. This precision enhances trial outcomes by grouping patients based on cognitive status and enables more personalized treatments. The framework is particularly relevant for genetically determined ALS and presymptomatic patients, for whom gene therapies are emerging. Building on concepts like mild motor impairment introduced by Benatar et al. [22], MBNI could extend to encompass cognitive and behavioral aspects, capturing ALS patients in prodromal phases. Recognizing these early signs enables better understanding of disease progression and therapeutic refinement.

Survival analysis further supports the clinical importance of early cognitive and behavioral changes. Significant differences in survival outcomes for MCI and MCBI patients challenge conventional views that cognitive impairments must be advanced to affect prognosis. Instead, our findings align with evidence that even mild impairments have substantial clinical implications.

However, a recent meta‐analysis on MBI [41] suggests that while MBI often precedes cognitive decline, it does not necessarily accelerate neurodegeneration in the same way as MCI, MCBI, or FTD. This distinction implies that although behavioral symptoms, such as apathy or disinhibition, may significantly affect quality of life, they do not necessarily contribute to the same degree of functional decline or mortality risk observed in cognitive impairment. This could, at least in part, explain the better survival observed in the MBI group. Conversely, cognitive impairment (MCI, MCBI, and FTD) may be associated with more extensive cortical dysfunction and a faster disease progression. Studies have shown that executive dysfunction and cognitive impairment correlate with reduced survival in ALS [10, 42], likely due to impaired decision‐making, reduced adherence to care, and increased neuroanatomical burden [10, 12, 42].

Notably, the overall prevalence of MCI, alone or combined with MBI, in our ALS cohort (48.8%) was higher than that reported in the general elderly Italian population (6%–21.6%) [43, 44, 45, 46], suggesting a disease‐related rather than an age‐related condition.

Moreover, the MBNI framework resonates with MCI criteria in other neurodegenerative diseases. As Abrahams [47] recently reviewed, ALS‐related cognitive impairments span domains like social cognition and memory, often neglected by Strong criteria's focus on executive function and language. A more comprehensive approach akin to MCI frameworks in Alzheimer's [48], Parkinson's [49] and in FTD [31, 50] could improve early detection of cognitive decline in ALS, enhancing patient outcomes.

Despite the strengths of our study, some limitations should be acknowledged. First, we did not gather data to assess the longitudinal evolution of cognitive and behavioral impairments in our sample. Second, while our classification approach provides a more sensitive stratification of patients, it lacks validation through neuroimaging or biomarker correlations. Finally, the present comprehensive neuropsychological assessment seems to provide relevant information, but its application in clinical practice would require sufficient time and patients' collaboration. A possible development could involve designing an abridged version to enhance feasibility.

In conclusion, early identification of cognitive/behavioral impairments highlights the continuum from normal cognition to FTD‐dem, capturing transitional phases of ALS. Recognizing a “mild” stage enhances understanding of ALS as a multisystem disorder and informs early interventions, particularly for genetic forms where innovative therapies could optimize treatment efficacy. Future research should validate MBNI across diverse ALS populations and explore its implications for personalized care, clinical trials, and therapeutic strategies.

## Author Contributions

**Myriam Spisto:** conceptualization, writing – review and editing, methodology, data curation, writing – original draft. **Pasquale Moretta:** conceptualization, validation, project administration, writing – review and editing. **Gianmaria Senerchia:** methodology, investigation, data curation. **Valentina Virginia Iuzzolino:** investigation, methodology, data curation. **Lucia Aruta:** investigation, methodology, data curation, formal analysis. **Elena Salvatore:** methodology, formal analysis, data curation. **Gabriella Santangelo:** conceptualization. **Luigi Trojano:** conceptualization, writing – original draft, writing – review and editing, formal analysis, software. **Raffaele Dubbioso:** conceptualization, investigation, writing – original draft, writing – review and editing, formal analysis, data curation, supervision, software, methodology.

## Ethics Statement

The study was approved by the ethics committee of the University Federico II of Naples (Protocol IDs 100/17/ES01 and 93/2023). Written informed consent was obtained from each patient or their guardian.

## Conflicts of Interest

The authors declare no conflicts of interest.

## Supporting information


Data S1.

Data S2.

## Data Availability

The dataset supporting the conclusions of the manuscript will be made available by the authors to any qualified researcher without breaching participant confidentiality.
