# Supplementary material for: Identifying Mild Behavioral and Neurocognitive Impairment in Amyotrophic Lateral Sclerosis (MBNI‐ALS) Provides Key Prognostic Insights
Source: Eur J Neurol. 2025 May 1;32(5):e70171. doi: 10.1111/ene.70171 (PMC12045931; doi:10.1111/ene.70171)
Supplement: Supplementary file 1 — Data S1. [file ENE-32-e70171-s001.docx]

**Supplementary data**

**Title:** Identifying Mild Behavioural and Neurocognitive impairment in Amyotrophic Lateral Sclerosis (MBNI-ALS) provides key Prognostic insights

**Authors:** Myriam Spisto^1^, PhD, Pasquale Moretta^3^, PhD, Gianmaria Senerchia^1^, MD, Valentina Virginia Iuzzolino^1^, MD, Lucia Aruta^1^, PhD, Elena Salvatore^4^, MD, PhD, Gabriella Santangelo^2^, PhD, Luigi Trojano^2^, MD, PhD, Raffaele Dubbioso^1*^, MD, PhD.

**Cognitive and behavioural evaluation**

After clinical evaluation, all participants underwent an extensive neuropsychological battery. A neuropsychologist with specific expertise in ALS assessment (M.S.) administered a multi-domain battery composed of Italian versions of standardized tests to assess the cognitive and behavioural profile of each patient. The multi-domain battery (Supplementary Table 1) assessed global cognitive functioning (MMSE, ECAS). A thorough assessment evaluated the main cognitive domains such as: Executive Functions (ECAS, SDMT, WCST, ST, FAS, CAT, FAB), Language (ECAS), Visuo-spatial Ability (ECAS, REYc, CLOCK, PM), Verbal (IR, DR, ECAS) and Visuo-spatial Memory (REYd).

The neuropsychological battery was designed to comprehensively assess cognitive functions typically affected in ALS, with a particular focus on executive functions, language, and visuospatial abilities. Multiple tests were used for executive functions to capture distinct subdomains (e.g., cognitive flexibility, working memory, inhibition). To ensure robustness and avoid overestimation of impairments, a diagnosis of executive dysfunction required deficits in at least two independent tests within the same domain.

Furthermore, behavioural changes usually associated with ALS were assessed through semi-structured (ECAS) and structured (FBI, Mild Behavioural Impairment Checklist, MBI-C) interviews with the caregiver.

Finally, all patients were assessed, net of disabling motor deficits, on their autonomy in basic daily living activities (ADL) and instrumental activities of daily living (IADL).

These tests were used to classify patients according to two principal frameworks: the Revised Diagnostic Criteria for ALS-frontotemporal spectrum disorder (ALS-FTDS) and the novel diagnostic approach borrowed from other neurodegenerative diseases such as Parkinson's disease (PD) and Alzheimer disease.

In the Revised Diagnostic Criteria for ALS-FTDS (or Strong classification) (Strong et al., 2017), participants were classified as cognitively normal (CN) if they showed no significant cognitive deficits, cognitively impaired (CI) if cognitive deficits were present, and as having behavioural impairment (BI) if significant behavioural changes were observed (Strong et al., 2017).

In the MBNI diagnostic approach, participants with cognitive impairment without significant functional decline were classified as MCI. MCI was defined by performance at least two standard deviations below the expected mean, adjusted for age and education. MCI single-domain involved abnormalities in two non-overlapping tests within a single cognitive domain, while MCI multiple-domain involved abnormalities in at least one test across two or more domains (Litvan et al., 2012). Behavioural changes such as apathy, emotional dysregulation, impulse control issues, social inadequacy, or abnormal thoughts/perceptions, assessed using the Mild Behavioural Impairment Checklist (MBI-C) (Ismail et al., 2017), were classified as mild behavioural impairment (MBI) (Ferraro et al., 2023). Patients exhibiting both mild cognitive and behavioural deficits were classified as having mild cognitive and behavioural impairment (MCBI).

Patients with evidence of progressive deterioration in behaviour and/or cognition were classified as having frontotemporal dementia (FTD) if they met criteria outlined by Rascovsky et al. (Rascovsky et al., 2011), including at least three behavioural or cognitive symptoms, or two symptoms along with loss of insight or psychotic features. Alternatively, patients with language disorders meeting criteria for semantic dementia or primary progressive aphasia (PPA), often coexisting with behavioural or cognitive symptoms, were also classified under FTD. Cases of ALS with dementia not fitting the typical ALS-FTD profile—such as Alzheimer's disease, vascular dementia, or mixed dementia (e.g., AD-vascular dementia)—were categorized as ALS-associated dementia (Strong et al., 2017). To ensure sufficient statistical power, these dementia subtypes were grouped into a single experimental category: FTD-dem.

The classifications of other dementias were made in accordance with the diagnostic criteria of the DSM: (1) Major neurocognitive disorder due to Alzheimer's disease: Gradual and progressive onset of decline in cognitive function in one or more cognitive domains (learning and memory) that affects independence in activities of daily living and the disturbance does not occur exclusively during a delirium and cannot be better explained by another mental disorder. (2) Vascular dementia: Evidence of cerebrovascular disease, i.e., significant cerebrovascular injury by history, physical examination, or neuroimaging. Abrupt or fluctuating cognitive decline in one or more cognitive domains that significantly interferes with independence in activities of daily living. The disturbance does not occur exclusively during a delirium and cannot be better explained by another mental disorder. (3) Mixed dementia: This condition refers to situations in which cognitive decline can be explained by more than one cause, usually a combination of Alzheimer's disease and vascular dementia. Summary for diagnostic criteria, the patient must meet criteria for both Alzheimer's disease and vascular dementia.

On average, the complete neuropsychological evaluation required approximately **60–75 minutes**, depending on the patient's condition and motor limitations.

Finally, to minimize the influence of motor impairments on test performance, alternative administration modalities were implemented. Verbal responses were permitted for tasks requiring manual execution, and written or pointing responses were allowed for patients with speech deficits. Extended response time was granted when necessary to ensure fair cognitive evaluation.

**Neuropsychological Assessment**

Following clinical evaluation, all participants underwent an extensive cognitive assessment, which included the Italian versions of the following tests:

- Mini-Mental State Examination (MMSE) for the assessment of global cognitive functioning (Folstein et al., 1975);
- Edinburgh Cognitive and Behavioural ALS Screen (ECAS), a rapid screening tool, which typically takes 15 to 20 minutes to complete and includes an ALS-specific section that assesses executive functions (reverse digit span, alternation test, sentence completion, and social cognition), verbal fluency, and language (naming, comprehension, spelling) with scores from 0 to 100 points. It has a non-ALS-specific section that assesses memory (immediate recall, delayed recall, and recognition) and visuospatial abilities, with scores from 0 to 36 points. The total ECAS score, therefore, ranges from 0, indicating the worst performance, to 136, representing the best performance (Siciliano et al., 2017);
- Frontal Assessment Battery (FAB) for a global assessment of prefrontal functions (Dubois et al., 2000);
- Wisconsin Card Sorting Test (WCST), for the assessment of the ability to process abstract categories, to modify the category according to the contingent situation and to inhibit imitation and perseveration (Laiacona et al., 2000);
- the interference task of the Stroop test (ST), for the assessment of the inhibition of automatic responses (Barbarotto et al., 1998);
- phonological (FAS) (Carlesimo et al., 1996) and semantic (CAT) (Novelli, 1986) verbal fluency tasks for the ability to scan the lexical store, cognitive flexibility and auditory-verbal working memory;
- Symbol Digit Modality Test (SDMT) (Langdon et al., 2012) for the assessment of sustained attention, vigilance and working memory;
- Clock Drawing Test (CLOCK) (Siciliano et al., 2016) and Raven’s 47 progressive colored matrices for visuo-spatial and planning functions (Carlesimo et al., 1996);
- Copy of the Rey-Osterrieth Complex Figure Test (REYc) for visuo-constructive abilities for complex material (Caffarra et al., 2002);
- Delayed recall of the Rey-Osterrieth Complex Figure Test (REYd) for the assessment of long-term visuo-spatial memory (Caffarra et al., 2002);
- Immediate and Delayed recall of Rey’s 15 words for the evaluation of verbal long-term memory (Carlesimo et al., 1996);

Furthermore, behavioural changes usually associated with ALS were assessed through short interviews with the caregiver:

- ECAS behavioural screening for the assessment of the presence of Behavioural Disinhibition, Apathy/Inertia, Loss of Sympathy/Empathy, Perseverance/Stereotype, Change in Eating Behaviour; score from 0 to 10 and psychotic symptoms (from 0 to 3) (Siciliano et al., 2017);
- The Frontal Behavioural Inventory (FBI) is a structured interview designed to assess behavioural and personality changes in patients with neurodegenerative conditions, particularly frontotemporal dementia. It evaluates 12 behavioural domains, including apathy, disinhibition, and compulsive behaviours, using a 0-3 severity scale (Alberici et al., 2007)
- Mild Behavioural Impairment (MBI) was assessed using the Italian version of the MBI-C, a 34-item questionnaire administered to caregivers and organized into five distinct domains. These domains include Apathy, which is assessed through six questions that assess cognitive, behavioural and emotional aspects; Affective/Emotional Dysregulation, which includes six items that assess low mood, anhedonia, despair, guilt, worry and panic; Impulse Control, composed of twelve questions that assess agitation, aggression, impulsivity, recklessness and anomalous rewards and reinforcements; Social Inadequacy, which addresses sensitivity, empathy, and tact through five questions; and Abnormal Thoughts/Perceptions, which assesses suspiciousness, grandiosity, and hallucinations through five additional questions. The MBI-C threshold value of 7.5 previously reported in the literature was used (Ferraro et al., 2023)

**Supplementary Table 1**

| **ABBREVIATION** | **FULL NAME** | **COGNITIVE DOMAINS ASSESSED** |
| --- | --- | --- |
| MMSE | Mini Mental State Examination | Global cognitive functioning |
| ECAS | Edinburgh Cognitive and Behavioural ALS Screen | Global cognitive functioning; Executive Functions;  Language;  Fluency;  Memory;  Visuo-spatial Ability;  Behavioural changes and psychotic symptoms |
| CLOCK | Clock Drawing Test | Visuo-spatial Ability |
| REYc | Copy of Rey-Osterrieth Complex Figure Test | Visuo-spatial Ability |
| PM | Raven’s 47 progressive colored matrices | Visuo-spatial Ability |
| FAS | Phonological Fluency | Executive Functions |
| CAT | Semantic Fluency | Executive Functions |
| ST | Stroop Test | Executive Functions |
| SDMT | Symbol Digit Modality Test | Executive Functions |
| WCST | Wisconsin Card Sorting Test | Executive Functions |
| FAB | Frontal Assessment Battery | Executive Functions |
| IR | Immediate recall of Rey’s 15 words | Verbal Long Term Memory |
| DR | Delayed recall of Rey’s 15 words | Verbal Long Term Memory |
| REYd | Delayed recall of Rey-Osterrieth Complex Figure Test | Visuo-spatial Long-Term Memory |
| FBI | Frontal Behavioural Inventory | Behavioural changes |
| MBI-C | Mild Behavioural Impairment Checklist | Behavioural changes |
| ADL | Basic daily living activities |  |
| IADL | Instrumental activities of daily living |  |

**Supplementary Table 2.** Characterization of Mild Cognitive and/or Behavioural Impairment in the Cognitively Normal (CN) ALS patients

| **CN (n=92)** | | | | | | | | | |
| --- | --- | --- | --- | --- | --- | --- | --- | --- | --- |
| **NC (n=30)** | **MCI (n=18)**  **Cognitive domains** | | | | **MBI (n=30)**  **Behavioural domains** | | | | |
|  | Single domain – memory (n) | | Single domain – visuospatial (n) | Multiple  Domains (n) | Apathy (mean ± sd) | Affective/ Emotional Dysregulation (mean ± sd) | Impulse Control (mean ± sd) | Social Inadequacy (mean ± sd) | Abnormal Thoughts/ Perceptions (mean ± sd) |
|  | 0 | | 2 | 16 | 4.8 ± 4.77 | 8.87 ± 4.13 | 5.53 ± 5.62 | 1.50 ± 2.78 | 0.70 ± 1.47 |
| **MCBI (n=14)** | | | | | | | | | |
|  | | **Cognitive domains** | | | **Behavioural domains** | | | | |
|  | Single domain – memory (n) | | Single domain - visuospatial(n) | Multiple  Domains (n) | Apathy (mean ± sd) | Affective/ Emotional Dysregulation (mean ± sd) | Impulse Control (mean ± sd) | Social Inadequacy (mean ± sd) | Abnormal Thoughts/ Perceptions (mean ± sd) |
|  | 1 | | 0 | 13 | 6.11 ±5.50 | 9.25 ± 5.44 | 7.38 ± 6.30 | 1.36 ± 2.52 | 0.69 ± 1.50 |

**Supplementary Table 3.**

**Univariate Cox Regression Analysis of Clinical and Cognitive Variables Predicting Survival in ALS Patients**

|  | **Univariate Analysis** | | | |
| --- | --- | --- | --- | --- |
| **Variables** | **Hazard Ratio** | **95% Confidence Interval** | | **p-value** |
| Age (years) | 1.049 | 1.026 – 1.072 | | **< 0.001** |
| Onset (reference: bulbar) | 0.29 | 0.18 – 0.48 | | **<0.001** |
| Education (years) | 0.924 | 0.88 – 0.97 | | **0.001** |
| Disease Duration (months) | 0.92 | 0.90 – 0.94 | | **<0.001** |
| Rate of Progression | 1.53 | 1.36 – 1.72 | | **<0.001** |
| King’s stages | 1.50 | 1.21 – 1.85 | | **<0.001** |
| MBNI classification |  |  | |  |
| NC | Reference | |  | |
| MCI | 6.746 | 1.909 – 23.839 | | **0.003** |
| MBI | 2.798 | 0.795 – 9.849 | | 0.109 |
| MCBI | 5.331 | 1.628 – 17.456 | | **0.006** |
| FTD-dem | 6.987 | 1.994 – 24.484 | | **0.002** |
| Strong Classification |  |  | |  |
| CN | Reference | |  | |
| CI | 2.088 | 1.142 – 3.816 | | **0.017** |
| BI | 1.274 | 0.576 – 2.815 | | 0.550 |
| CBI | 1.888 | 0.951 – 3.749 | | 0.069 |
| FTD-dem | 2.357 | 1.243 – 4.467 | | **0.009** |

In bold significant *p* < 0.05.

ALSFRS-R: Amyotrophic Lateral Sclerosis Functional Rating Scale - Revised; BI: Behavioural Impairment; CBI: Cognitive and Behavioural Impairment; CI: Cognitive Impairment; CN: Cognitively Normal; FTD-dem: Frontotemporal Dementia and other dementias; MBI: Mild Behavioural Impairment; MCBI: Mild Cognitive and Behavioural Impairment; MCI: Mild Cognitive Impairment; NC: Normal Cognition.

**Supplementary Figure 1**

**Cognitive and Behavioural Impairments in Patients Classified as Cognitively Normal (CN) by the Strong Criteria**


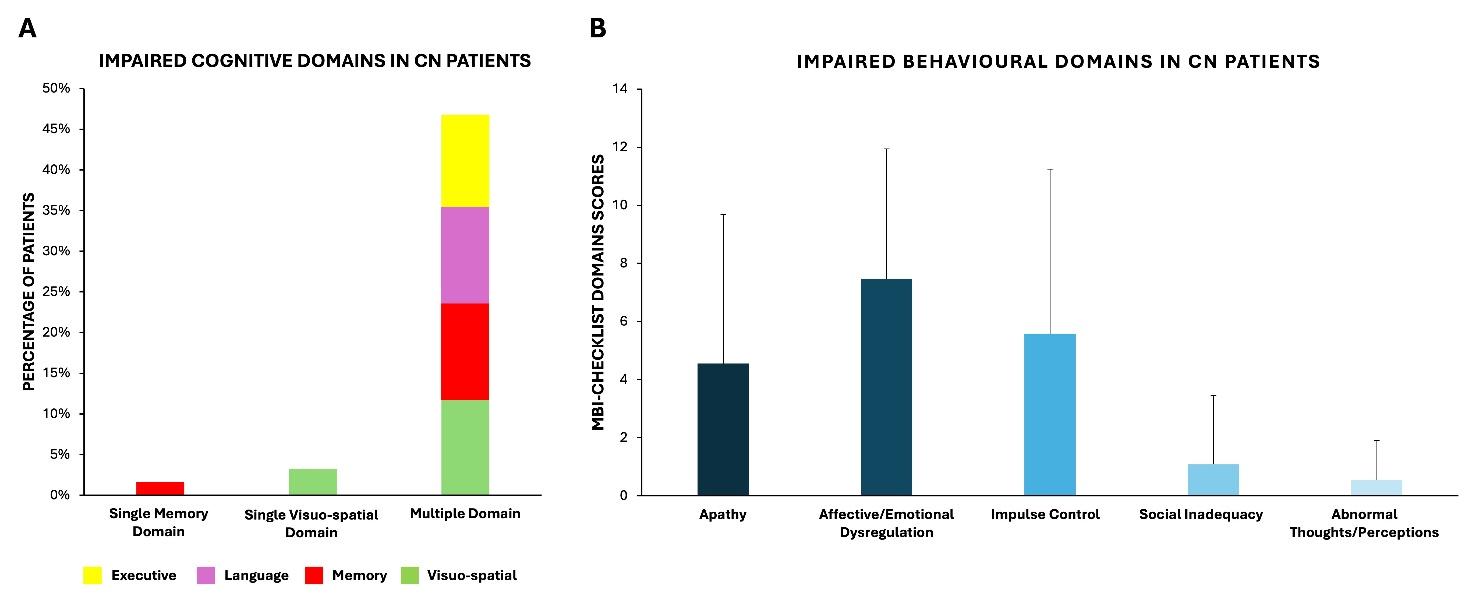


In Panel A, the percentage of patients exhibiting impairments across specific cognitive domains is displayed. The graph highlights single-domain impairments, including memory (red) and visuo-spatial function (green), as well as multiple-domain impairments. Executive function impairments are marked in yellow, and language impairments in purple. The data indicates that most patients show impairments across multiple cognitive domains rather than isolated deficits.

In Panel B, the mean scores from the MBI-checklist are presented, detailing impairments across behavioural domains. Apathy and affective/emotional dysregulation exhibit the highest mean scores, followed by impulse control issues. Behavioural changes related to social inadequacy and abnormal thoughts/perceptions are less frequently reported, reflecting a lower prevalence in this cohort.

**References**

Alberici, A., Geroldi, C., Cotelli, M., Adorni, A., Calabria, M., Rossi, G., Borroni, B., Padovani, A., Zanetti, O., & Kertesz, A. (2007). The Frontal Behavioural Inventory (Italian version) differentiates frontotemporal lobar degeneration variants from Alzheimer’s disease. *Neurological Sciences*, *28*(2), 80–86. https://doi.org/10.1007/s10072-007-0791-3

Barbarotto, R., Laiacona, M., Frosio, • R, Vecchio, • M, Farinato, A., & Capitani, • E. (1998). A normative study on visual reaction times and two Stroop colour-word tests. In *Ital J Neurol Sci* (Vol. 19).

Caffarra, P., Vezzadini, G., Dieci, F., Zonato, F., & Venneri, A. (2002). Rey-Osterrieth complex figure: normative values in an Italian population sample. *Neurological Sciences*, *22*(6), 443–447. https://doi.org/10.1007/s100720200003

Carlesimo, G. A., Caltagirone, C., Gainotti, G., Fadda, L., Gallassi, R., Lorusso, S., Marfia, G., Marra, C., Nocentini, U., & Parnetti, L. (1996). The Mental Deterioration Battery: Normative Data, Diagnostic Reliability and Qualitative Analyses of Cognitive Impairment. *European Neurology*, *36*(6), 378–384. https://doi.org/10.1159/000117297

Dubois, B., Slachevsky, ; A, Litvan, ; I, & Pillon, B. (2000). *The FAB A frontal assessment battery at bedside*. https://www.neurology.org

Ferraro, P. M., Gervino, E., De Maria, E., Meo, G., Ponzano, M., Pardini, M., Signori, A., Schenone, A., Roccatagliata, L., & Caponnetto, C. (2023). Mild behavioral impairment as a potential marker of predementia risk states in motor neuron diseases. *European Journal of Neurology*, *30*(1), 47–56. https://doi.org/10.1111/ene.15570

Folstein, M. F., Folstein, S. E., & Mchugh, P. R. (1975). “MINI-MENTAL STATE” A PRACTICAL METHOD FOR GRADING THE COGNITIVE STATE OF PATIENTS FOR THE CLINICIAN*. In *J. gsychiaf. Res* (Vol. 12). Pergamon Press.

Ismail, Z., Agüera-Ortiz, L., Brodaty, H., Cieslak, A., Cummings, J., Fischer, C. E., Gauthier, S., Geda, Y. E., Herrmann, N., Kanji, J., Lanctôt, K. L., Miller, D. S., Mortby, M. E., Onyike, C. U., Rosenberg, P. B., Smith, E. E., Smith, G. S., Sultzer, D. L., & Lyketsos, C. (2017). The Mild Behavioral Impairment Checklist (MBI-C): A Rating Scale for Neuropsychiatric Symptoms in Pre-Dementia Populations. *Journal of Alzheimer’s Disease*, *56*(3), 929–938. https://doi.org/10.3233/JAD-160979

Laiacona, M., Inzaghi, M. G., De Tanti, A., & Capitani, E. (2000). Wisconsin card sorting test: a new global score, with Italian norms, and its relationship with the Weigl sorting test. *Neurological Sciences*, *21*(5), 279–291. https://doi.org/10.1007/s100720070065

Langdon, D. W., Amato, M. P., Boringa, J., Brochet, B., Foley, F., Fredrikson, S., Hämäläinen, P., Hartung, H. P., Krupp, L., Penner, I. K., Reder, A. T., & Benedict, R. H. B. (2012). Recommendations for a brief international cognitive assessment for multiple sclerosis (BICAMS). In *Multiple Sclerosis Journal* (Vol. 18, Issue 6, pp. 891–898). https://doi.org/10.1177/1352458511431076

Litvan, I., Goldman, J. G., Tröster, A. I., Schmand, B. A., Weintraub, D., Petersen, R. C., Mollenhauer, B., Adler, C. H., Marder, K., Williams‐Gray, C. H., Aarsland, D., Kulisevsky, J., Rodriguez‐Oroz, M. C., Burn, D. J., Barker, R. A., & Emre, M. (2012). Diagnostic criteria for mild cognitive impairment in Parkinson’s disease: *Movement* Disorder Society Task Force guidelines. *Movement Disorders*, *27*(3), 349–356. https://doi.org/10.1002/mds.24893

Novelli, G. ; P. C. ; C. E. ; L. M. ; V. G. C. S. (1986). Tre test clinici di ricerca e produzione lessicale. Taratura su soggetti normali. *Archivio Di Psicologia, Neurologia e Psichiatria*, *47*(4), 477–506.

Rascovsky, K., Hodges, J. R., Knopman, D., Mendez, M. F., Kramer, J. H., Neuhaus, J., Van Swieten, J. C., Seelaar, H., Dopper, E. G. P., Onyike, C. U., Hillis, A. E., Josephs, K. A., Boeve, B. F., Kertesz, A., Seeley, W. W., Rankin, K. P., Johnson, J. K., Gorno-Tempini, M. L., Rosen, H., … Miller, B. L. (2011). Sensitivity of revised diagnostic criteria for the behavioural variant of frontotemporal dementia. *Brain*, *134*(9), 2456–2477. https://doi.org/10.1093/brain/awr179

Siciliano, M., Santangelo, G., D’Iorio, A., Basile, G., Piscopo, F., Grossi, D., & Trojano, L. (2016). Rouleau version of the Clock Drawing Test: age- and education-adjusted normative data from a wide Italian sample. *Clinical Neuropsychologist*, *30*, 1501–1516. https://doi.org/10.1080/13854046.2016.1241893

Siciliano, M., Trojano, L., Trojsi, F., Greco, R., Santoro, M., Basile, G., Piscopo, F., D’Iorio, A., Patrone, M., Femiano, C., Monsurrò, M., Tedeschi, G., & Santangelo, G. (2017). Edinburgh Cognitive and Behavioural ALS Screen (ECAS)-Italian version: regression based norms and equivalent scores. *Neurological Sciences*, *38*(6), 1059–1068. https://doi.org/10.1007/s10072-017-2919-4

Strong, M. J., Abrahams, S., Goldstein, L. H., Woolley, S., Mclaughlin, P., Snowden, J., Mioshi, E., Roberts-South, A., Benatar, M., HortobáGyi, T., Rosenfeld, J., Silani, V., Ince, P. G., & Turner, M. R. (2017). Amyotrophic lateral sclerosis - frontotemporal spectrum disorder (ALS-FTSD): Revised diagnostic criteria. *Amyotrophic Lateral Sclerosis and Frontotemporal Degeneration*, *18*(3–4), 153–174. https://doi.org/10.1080/21678421.2016.1267768
